# Natural Variants of *C. elegans* Demonstrate Defects in Both Sperm Function and Oogenesis at Elevated Temperatures

**DOI:** 10.1371/journal.pone.0112377

**Published:** 2014-11-07

**Authors:** Lisa N. Petrella

**Affiliations:** 1 Department of Molecular, Cell and Developmental Biology, University of California Santa Cruz, Santa Cruz, California, United States of America; 2 Department of Biological Sciences, Marquette University, Milwaukee, Wisconsin, United States of America; University of North Carolina, United States of America

## Abstract

The temperature sensitivity of the germ line is conserved from nematodes to mammals. Previous studies in *C. briggsae* and *Drosophila* showed that isolates originating from temperate latitudes lose fertility at a lower temperature than strains originating from tropical latitudes. In order to investigate these relationships in *C. elegans,* analysis of the fertility of 22 different wild-type isolates of *C. elegans* isolated from equatorial, tropical and temperate regions was undertaken. It was found that there are significant temperature, genotype and temperature × genotype effects on fertility but region of isolation showed no significant effect on differences in fertility. For most isolates 100% of the population maintained fertility from 20°C to 26°C, but there was a precipitous drop in the percentage of fertile hermaphrodites at 27°C. In contrast, all isolates show a progressive decrease in brood size as temperature increases from 20°C to 26°C, followed by a brood size near zero at 27°C. Temperature shift experiments were performed to better understand the causes of high temperature loss of fertility. Males up-shifted to high temperature maintained fertility, while males raised at high temperature lost fertility. Down-shifting males raised at high temperature generally did not restore fertility. This result differs from that observed in *Drosophila* and suggested that in *C. elegans* spermatogenesis or sperm function is irreversibly impaired in males that develop at high temperature. Mating and down-shifting experiments with hermaphrodites were performed to investigate the relative contributions of spermatogenic and oogenic defects to high temperature loss of fertility. It was found that the hermaphrodites of all isolates demonstrated loss in both spermatogenic and oogenic germ lines that differed in their relative contribution by isolate. These studies uncovered unexpectedly high variation in both the loss of fertility and problems with oocyte function in natural variants of *C. elegans* at high temperature.

## Introduction

The maintenance of germline function is necessary for population survival. This maintenance is challenged when organisms experience temperature fluctuations in their environment. Germline function is highly sensitive to temperature. Even small increases of a few degrees Celsius can result in a population that goes from fertile to sterile. Complete loss of germline function at a temperature that does not stress non-germline tissues is observed across a range of organisms[1]–[4]. This is exemplified by the fact that the testes of many mammals are exterior to the body to allow for a lower thermal zone for spermatogenesis to take place. For invertebrates that cannot control their internal temperatures this is an even greater challenge. Recent work has shown that *Caenorhabditis* nematodes in actively reproducing populations do not live deep within soils where temperatures are more likely to be buffered. Instead, they reproduce in rotting fruits and other plant matter at the surface [5], [6]. Thus, these nematodes are likely to experience temperatures within the range that result in sterile individuals. *C. elegans* fertility is highest around 20°C and slowly declines as temperature increases until sterility is reached at 27°C [7]. *C. elegans* is a cosmopolitan species that is distributed throughout the world and is found in locations where it would experience temperatures outside of this fertile zone (Table 1).

**Table 1:** Summary of isolate information used in this study.

Isolate	Location of origin	Latitude	Approximate elevation (m)	Average summer temperature (°C, High, Low)	Average winter temperature (°C, High, Low)
MY1	Lingen, Germany	52°54′N	24	23, 12^C^	4, −1.7^C^
MY2	Roxel, Germany	51°96′N	77	23, 12^C^	4, −1.7^C^
MY3	Roxel, Germany	51°96′N	77	23, 12^C^	4, −1.7^C^
N2	Bristol, UK	51°45′N	18	21, 12^A^	7, 2 ^A^
CB4932	Tauton, UK	51°02′N	38	21, 12^B^	5, 1.6^A^
TR389	Madison, WI	43°04′N	267	27.8, 16.1	−3.8, −12.6
JU1172	Concepcion, Chile	36°87′S	28	23, 13^I^	11, 5^I^
JU1171	Concepcion, Chile	36°82′S	28	23, 13^I^	11, 5^I^
JU1652	Montevideo, Ur	34°86′S	16	28, 16.7	14.4, 6.1
DR1350	Pasadena, CA	34°13′N	256	31.0, 16.2	19.9, 6.8
JU1088	Kakegawa, Japan	34°76′N	38	28, 21^K^	8.3, −1.6
AB3	Adelaide, AU	34°93′S	44	30, 16	15, 7
CB4507	Palm Canyon, CA	33°47′N	122	40.0, 24.4^#^	21.3, 6.8^#^
ED3046	Ceres, South Africa	33°22′S	478*	26, 16^F^	17, 7^F^
JU258	Ribeiro Frio, Madeira	32°73′N	1117	29, 22^E^	19, 13^E^
ED3040	Johannesburg, South Africa	26°10′S	1669*	27, 16^D^	19, 3^D^
CB4856	Hawaii, USA*	21°33′N		30.6, 22.4^J^	27.2, 18.1^J^
DL238	Manuka, HI USA	19°22′N	584	23.9, 13.9^L^	25.6, 17.2
LKC34	Madagascar*	18°00S		26, 16^H^	20, 9^H^
DL200	Addis Ababa, Ethiopia	9°03′N	2390	20.6/13.6	23.8/6.1
ED3077	Nairobi, Kenya	1°19′S	1708*	25, 12^G^	21, 11^G^
ED3072	Limuru, Kenya	1°05′S	2356*	25, 12^G^	21, 11^G^

For Non-USA locations average temperature for closest city listed in US Dept. of Commerce 1991.

Winter Temp: July Southern Hemisphere, January Northern Hemisphere;

Summer Temp: January Southern Hemisphere, July Northern Hemisphere.

*Exact location of isolation unknown.

The molecular pathways and structural components that are necessary for germline development and function in *C. elegans* have been studied extensively. However, this work has primarily been performed at temperatures below the thermal threshold of fertility. Additionally, these studies have been done almost exclusively in the background of the common lab strain of *C. elegans,* N2. This strain was initially isolated in Bristol, England over 50 years ago and has been demonstrated to show some laboratory adaptation [8]. Work in *Drosophila* and another *Caenorhabditis* species, *C. briggsae,* has shown that wild-type isolates of a species from differing latitudes showed distinct differences in thermal thresholds for loss of fertility [4], [9]. In both species, isolates from tropical latitudes show a thermal threshold for loss of fertility 1–2 degrees above that of isolates from temperate latitudes [2], [4], [9]. Previous work looking at the thermal range of *C. elegans* wild-type isolates demonstrated no origin × environment interaction such that isolates from higher latitudes did not lose fertility at temperatures lower than isolates from lower latitudes [3]. However, this study did not look at any isolates that originated from tropical latitudes nor at temperatures near the thermal threshold for loss of fertility, both of which are necessary to see clear differences in isolates of *C. briggsae.* Thus, any origin × environment effect on fertility at higher temperatures in *C. elegans* may have gone undetected.

Here the high temperature thermal threshold for loss of fertility in 22 different wild-type isolates of *C. elegans* from equatorial to temperate latitudes and from 5 continents and 4 islands (Table 1) was investigated. It was found that even when using tropical isolates and higher temperature regimes, there is no trend of origin × environment interactions. However, there are significant genotype × environment differences such that some isolates are much more thermal tolerant and others are much more thermal sensitive. It was also investigated whether these trends hold true for the uncommon male of the species, and it was found that males also have high temperature thermal sensitivity and showed varying amounts of recovery after heat exposure. Finally, the contribution of defects in sperm vs. the oogenic germ line in hermaphrodites was analyzed. Data showed that both contribute to loss of fertility at the thermal threshold to varying degrees in hermaphrodites of different isolates. This work demonstrates that loss of fertility at high temperature in *C. elegans* nematodes is a complex trait that is a result of multiple dysfunctions in the germ line of both sexes.

## Results

### Hermaphrodite fertility is affected by temperature and genetic background

Organisms generally show a temperature sensitive loss of fertility at temperatures that do not kill them. I wanted to investigate whether different wild-type isolates of *C. elegans* showed different thermal thresholds for loss of fertility and whether this was correlated with their latitude of origin. Previous work in *C. elegans* had shown no correlation between fertility rates of different *C. elegans* natural wild-type isolates and their latitude of origin when using isolates from temperate latitudes [3]. However, Harvey and Viney measured changes in fertility only at temperatures up to 25°C, where brood size is still relatively robust. The temperature at which the canonical wild-type strain N2 has been reported to lose the majority of fertility is 27°C [10]. Therefore, in this study the rates of fertility in 22 different wild-type isolates from 20°C to 27°C were investigated (Table 1). Two isolates, JU258 and MY1, were eliminated from analysis: JU258 because of constitutive dauer formation at 27°C and MY1 because all F1 embryos failed to hatch at 27°C. All further analysis was done on the remaining 20 isolates.

**Figure 1:**
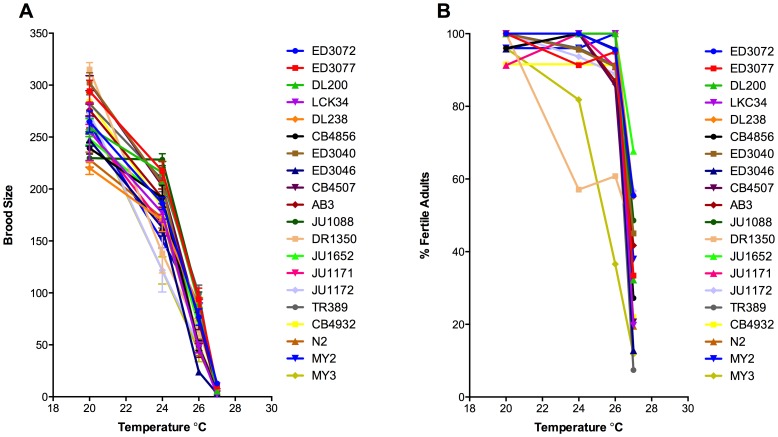
Increasing temperatures reduces brood size with a threshold for population fertility at 27°C. Mean brood size (A) and percentage of population that is fertile (B) graphed as a function of temperature. For all isolates as temperature increases brood size gradually decreases, while for most isolates the percentage of the population that is fertile does not decrease until 27°C. Isolates are arranged from most equatorial (ED3072) to least equatorial (MY3). Error bars indicate +1 SEM.

**Figure 2:**
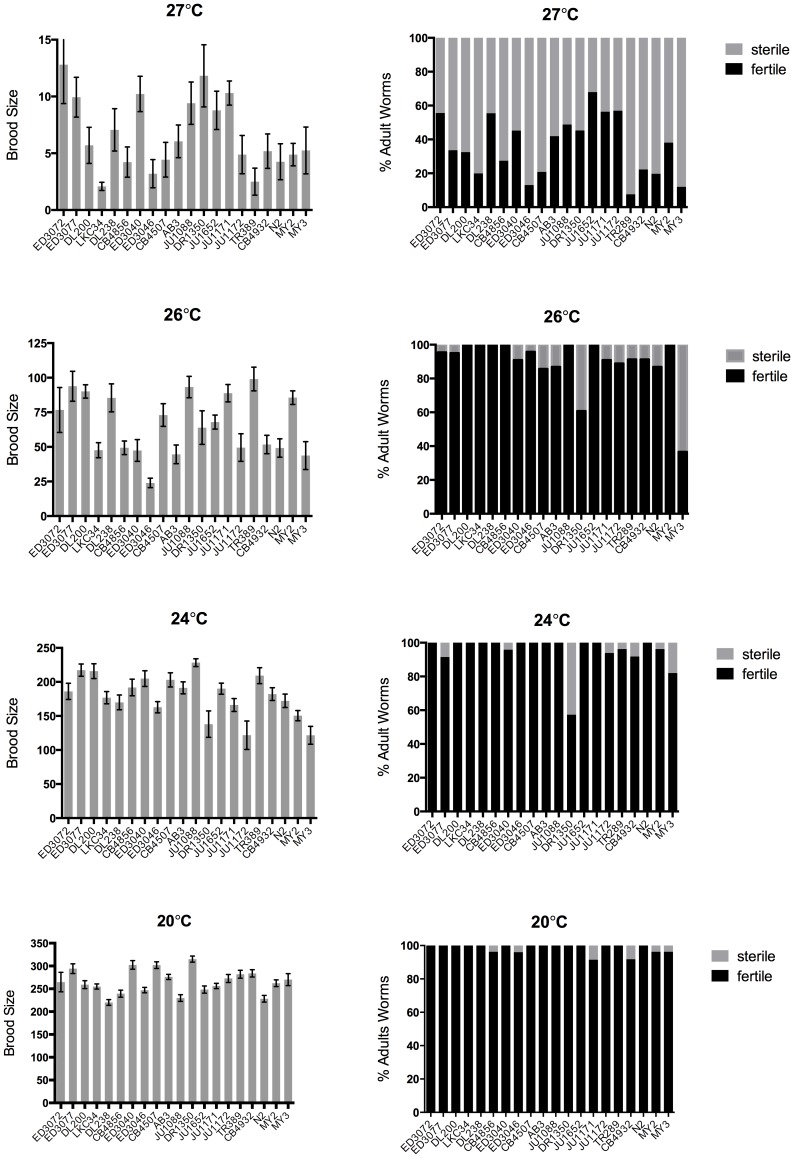
Individual isolates show different brood sizes at all temperatures. Mean brood size (left) and percentage of population that is fertile (right) of individual isolates are graphed at 20°C, 24°C, 26°C and 27°C. The level of lifetime brood size shows differences between isolates at all temperatures. Differences in the percentage of the population that is fertile are seen primarily at higher temperatures (26°C and 27°C). Isolates are arranged from most equatorial (ED3072) to least equatorial (MY3). Error bars indicate +1 SEM.

**Figure 3:**
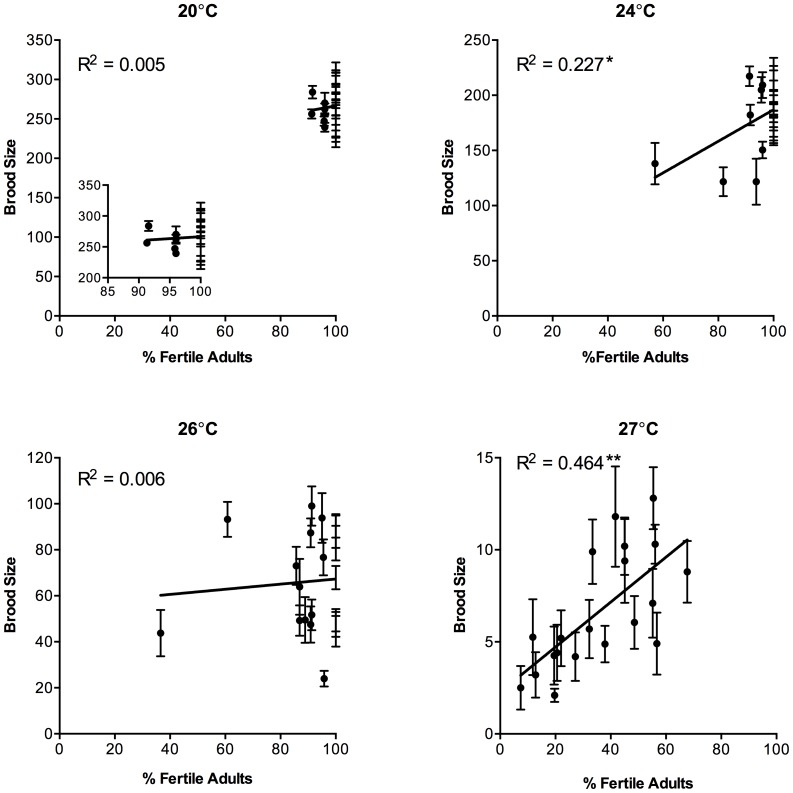
Isolate brood size and percentage of fertile adults are correlated at 27°C. Mean brood size of individual isolates correlated with percentage of the population that is fertile of the same isolate at 20°C, 24°C, 26°C and 27°C. There is weak correlation between isolates that show a larger brood size with those that show a higher percentage of the population that is fertile at 24°C (*P* = 0.0338) and a strong correlation at 27°C (*P* = 0.001). Error bars indicate +1 SEM of brood size.

In order to fully assess the level of reproduction, both the percentage of the population that were able to produce offspring (% Fertile Adults) and the number of offspring that fertile individuals produced (Brood Size) were examined (Figure 1, Figure 2, Table 2). All isolates demonstrated the highest level of progeny production at 20°C and showed decreasing brood sizes as temperatures were increased (Figure 1A and Figure 2). In contrast, for the majority of isolates, there was very little loss in the percentage of hermaphrodites in the population that were fertile until 27°C (Figure 1B and Figure 2). Because the level of population fertility was so stable in the majority of isolates, there was no significant correlation between the level of population fertility and average brood size at 20°C and 26°C (Figure 3). At 24°C there was a weak correlation between population fertility and average brood size (*R^2^* = 0.227, *P* = 0.03), which was due predominately to the loss of population fertility of a single isolate, DR1350. On the other hand, there was a strong correlation between the reduced number of hermaphrodites that were fertile and average brood size at 27°C (*R^2^* = 0.464, *P* = 0.001) (Figure 3).

**Table 2:** Summary of brood sizes from hermaphrodites at continuous temperature.

	Brood Size (SEM)			
	20°C	24°C	26°C	27°C
ED3072	264.79(10.27)	186.13(5.74)	76.71(7.79	12.80(1.69)
ED3077	294.23(10.57)	217.33(8.97)	93.84(10.81)	9.90(1.75)
DL200	259.04(8.64)	215.81(10.91)	90.09(4.79)	5.70(1.59)
LKC34	255.08(27.49)	176.91(8.97)	47.59(5.44)	2.10(0.36)
DL238	220.04(6.17)	169.91(10.89)	85.43(10.08)	7.10(1.87)
CB4856	239.39(5.61)	191.64(12.21)	49.35(4.89)	4.20(1.32)
ED3040	302.48(9.44)	204.91(11.49)	47.50(7.87)	10.20(1.56)
ED3046	247.17(5.86)	162.88(8.21)	23.96(3.44)	3.20(1.24)
CB4507	301.92(7.27)	203.04(10.38)	73.00(8.33)	4.40(1.53)
AB3	276.13(5.69)	191.29(8.80)	44.60(6.70)	6.05(1.44)
JU1088	229.83(2.26)	228.29(5.80)	93.26(7.60)	9.40(2.28)
DR1350	315.08(6.66)	138.00(18.88)	63.90(12.14)	11.80(2.73)
JU1652	248.35(5.75)	190.08(3.91)	67.92(5.08)	8.80(1.68)
JU1171	256.33(5.85)	166.17(9.46)	87.35(6.29)	10.30(1.06)
JU1172	272.67(8.89)	121.73(20.89)	49.50(9.96)	4.90(1.69)
TR389	282.00(8.65)	209.18(11.69)	99.00(8.58)	2.50(1.19)
CB4932	283.86(7.97)	182.18(9.35)	51.71(6.67)	5.20(1.52)
N2	228.21(7.26)	172.25(9.80)	49.20(6.61)	4.25(1.58)
MY2	262.38(7.31)	150.43(7.44)	85.62(4.81)	4.88(0.99)
MY3	270.17(13.13)	121.67(12.09)	43.75(10.09)	5.25(2.06)

**Figure 4:**
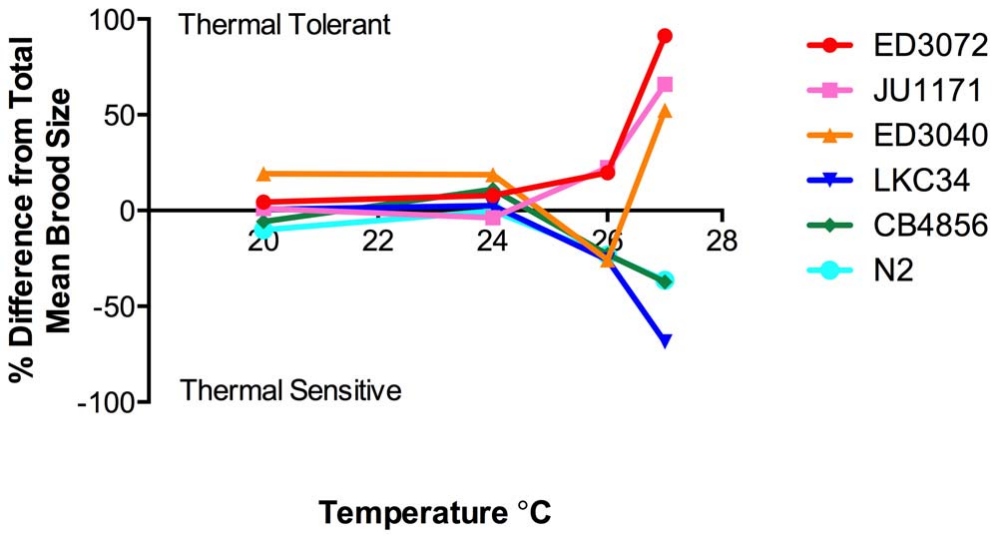
Thermal tolerant and thermal sensitive isolates can be distinguished at 27°C. A comparison of the mean brood size of individual isolates across the four temperatures with the combined mean brood size of all isolates at each temperature demonstrates differences among the brood size of isolates at 27°C. Three isolates (ED3072, JU1171 and ED3040) are more thermal tolerant than the average isolate while three isolates (LKC34, CB4856 and N2) are more thermal sensitive.

Along with a trend towards decreased brood size with increasing temperatures, it was observed that there are differences in brood size between isolates at individual temperatures. A multiple regression model was developed that explained 93% of the variance of brood size (*F_20,1630_* = 612.4, *P*<0.0001), with significant effects of temperature (*P*<0.0001), genotype (*P*<0.0001) and a genotype × temperature interaction (*P*<0.0001). Including latitude of origin, region of origin (Equatorial, Tropical and Temperate), haplotype (based on Andersen et al., 2011), and average summer high temperature at location of origin or average winter high temperature at location of origin did not strengthen the model. Post-hoc analysis of brood size among isolates at each temperature identified significant differences at all temperatures assayed, 20°C (*F_19,442_* = 7.884, *P*<0.001), 24°C (*F_19,420_* = 6.929, *P*<0.0001), 26°C (*F_19,369_*, *P*<0.0001), 27°C (*F_19,359_* = 4.603, *P*<0.0001). At 27°C three isolates, ED3072, ED3040 and JU1171, showed significantly higher brood sizes than three other isolates, LKC34, CB4856 and N2 (Figure 4 and Table 2). The three isolates that have higher brood size were designated as thermal tolerant and the three isolates with lower brood size as thermal sensitive. These groupings were not seen among specific isolates at lower temperatures. Interestingly, thermal tolerant isolates are from equatorial, tropical, and temperate latitudes, while thermal sensitive isolates are from tropical and temperate latitudes (Table 1).

### Male *C. elegans* wild-type isolates demonstrate loss of fertility at high temperature

To date, all experiments that examine temperature sensitivity in *Caenorhabditis* nematodes have been performed with hermaphrodites [3], [4], [10]. Because hermaphrodites produce both sperm and oocytes, any observed effects on fertility could be due to changes in the production or function of either gamete or the interaction of the two. In most other species investigated, specifically Drosophilids and mammals, the sperm are the primary temperature sensitive gamete [1], [2]. Therefore, the temperature sensitivity of male *Caenorhabditis* nematodes, which only produce sperm, was investigated. This analysis would begin to tease apart if sperm, oocytes or both are temperature sensitive in *C. elegans*.

**Figure 5:**
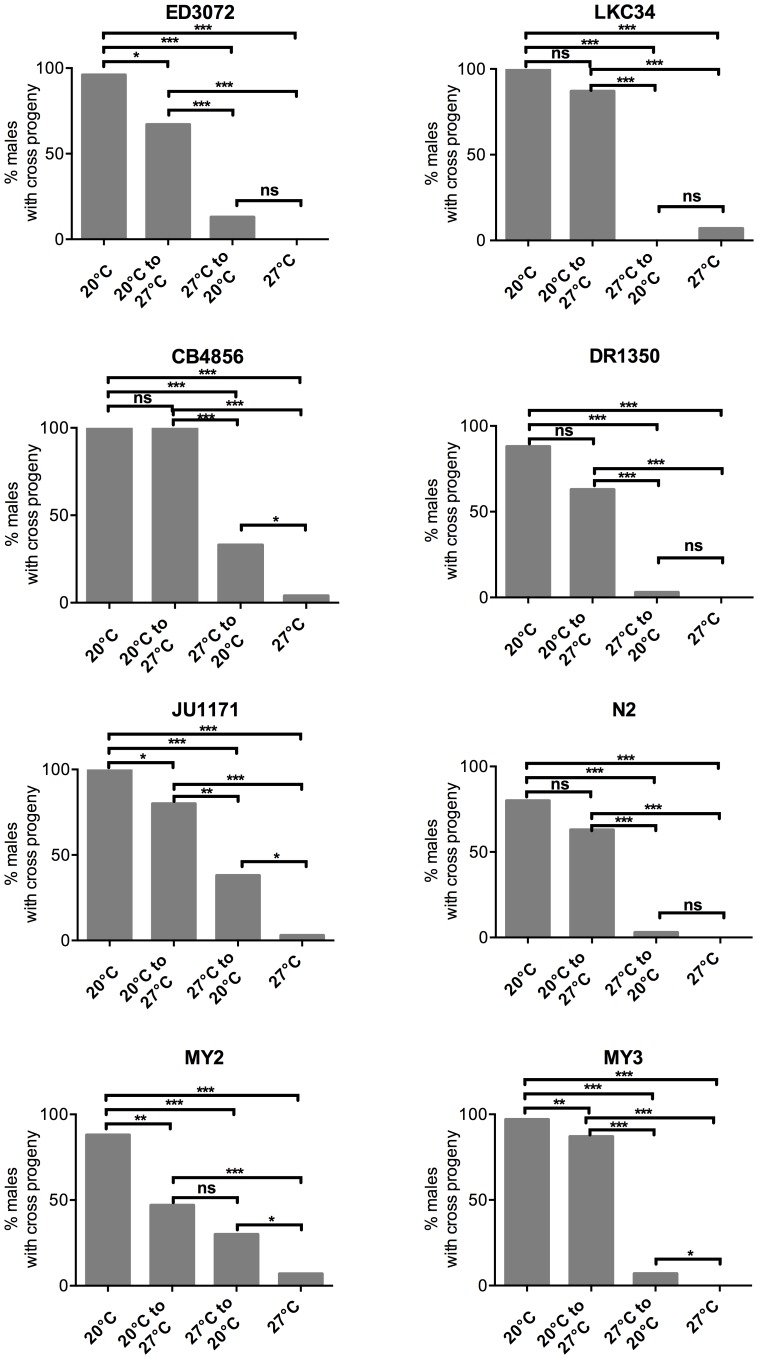
Male worms lose fertility at 27°C and rarely regain fertility upon down-shifting. Percentage of males that were able to produce cross-progeny in the population of eight isolates was assayed. Rearing temperature and temperature-shift at the L4/young adult stage of the males is indicated on the x-axis. Up-shifting males from 20°C to 27°C sometimes results in a small but significant reduction in fertility (ED3072, JU1171, MY2, MY3). Rearing and mating at 27°C results in a complete or close to complete loss of fertile males. Down-shifting from 27°C to 20°C occasionally results in a weak recovery of fertility that is significantly higher than the fertility at 27°C but always lower that the fertility if rearing is done at 20°C. Fisher exact test with significance indicated at *P<0.05, ** P<0.01, ***P<0.001, ns =  not significant.

The ability of males to produce cross progeny with hermaphrodites was scored under four different temperature regimes (Figure S1). All hermaphrodites were raised to the young adult stage at 20°C to reduce temperature effects on the hermaphrodite germ line. Eight isolates were analyzed that represented a range of brood sizes at high temperature as seen in hermaphrodites. In all isolates the majority of male worms (80–100%) were able to successfully have progeny when both raised at and mated at 20°C (Figure 5, Table 3). The majority of isolates (5/8) showed no significant loss in fertility rate when male worms raised at 20°C were up-shifted to 27°C at the young adult stage and allowed to mate. For 3 isolates (MY2, ED3072, and JU1171) there was a significant (*P*≤0.05) reduction in the ability to produce cross-progeny when up-shifted; however many (between 47–80%) of the males were still able to produce cross progeny under these conditions. This is in stark contrast to males that were both raised and allowed to mate at 27°C. Compared with either up-shifted or 20°C maintained males, males raised and mated at 27°C had a significant reduction in the ability to produce cross progeny. Four isolates (ED3072, DR1350, N2 and MY3) showed complete loss of male fertility at 27°C and the highest percentage of males of any isolate able to produce cross progeny was 7% (LKC34, and MY2) (Figure 5, Table 3). This is in contrast to the lowest percentage of males that can produce cross progeny in the other two experimental conditions being 47%.

**Table 3:** Summary of fertility rates in males in temperature-shift experiments.

	% Males with cross-progeny			
Isolate	20°C	20°C to 27°C	27°C to 20°C	27°C
ED3072	96	66.6	12.5	0
LKC34	100	86.7	0	6.7
CB4856	100	100	33.33	4.2
DR1350	88	63.3	3.3	0
JU1171	100	80	37.9	3.3
N2	80	63.3	3.3	0
MY2	88	46.7	30	6.7
MY3	96.7	86.7	6.7	0

Spermatogenesis in males continues throughout the lifetime of the organism. Because of this attribute male species of Drosophilids and mammals are able to recover fertility when down-shifted from a stressful temperature to a permissive temperature [9], [11]. To determine whether the loss of fertility of male *C. elegans* was able to recover, male worms were raised at 27°C and then down-shifted as young adults to 20°C and provided with 20°C raised hermaphrodites. To ensure that any lack of cross progeny was not due to the inability of a hermaphrodite to receive newly derived healthy sperm after receiving non-functional sperm, males were moved to plates with new hermaphrodites on day 3 (this was done in all temperature regimes tested). Of the eight isolates tested only three isolates (DR1350, JU1171 and MY2) showed a significant increase in the percentage of males that were able to produce cross-progeny compared to those same isolates both raised and maintained at 27°C (Figure 5, Table 3). All isolates raised at 27°C, then down-shifted to 20°C, had significantly fewer males that could successfully produce cross-progeny when compared with the same isolate raised at 20°C and then either mated at 20°C or 27°C. The sole exception was MY2, which showed no statistical difference between males raised at 20°C and mated at 27°C compared to males raised at 27°C and mated at 20°C (Figure 5, Table 3). These data indicate that there was variation in the ability of males of an isolate to recover upon down-shifting and that the recovery in those isolates that did recover was relatively weak.

### Hermaphrodite high temperature loss of fertility is due to a defect in both spermatogenic and oogenic germline function

*C. elegans* hermaphrodites make sperm only during the L4 larval stage. They then terminally switch to making oocytes when they molt to the adult stage. Because hermaphrodites have both sperm and oocyte, loss of progeny production at high temperature could be due to a loss of function in either gamete's development or function, or in both pathways. To distinguish between these possibilities temperature shift experiments paired with mating of hermaphrodites to same isolate of males were used (Figure S2). All males used in these experiments were raised at 20°C to ensure that any effect seen would be the result of the temperature treatment on the hermaphrodite. Temperature shifts were performed at the early adult stage so that oogenesis would have already begun.

**Figure 6:**
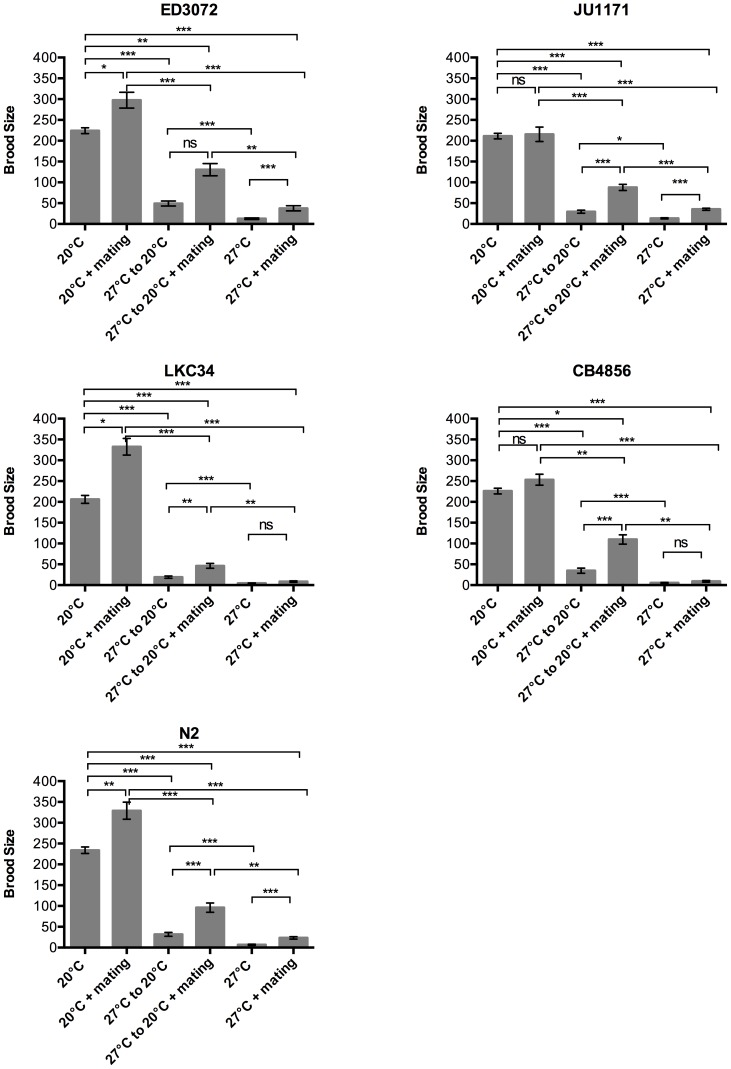
Hermaphrodites show deficiencies in both sperm function and oogenic germline function at 27°C. Brood size of only self-progeny or a hermaphrodite mated with a same isolate male was measured after 96 hours post-adult molt. Rearing temperature and temperature-shift at the young adult stage of the hermaphrodites along with mating status is indicated on x-axis. Hermaphrodites raised and mated with males at 27°C show a small but significant increase in brood size in 3/5 isolates. Down-shifting from 27°C at the young adult stage can also only result in a weak recovery of brood size as compared with worms raised at 27°C, that can only partial be enhanced by providing fresh non-heat exposed sperm by mating with males. All males used were raised until the L4/young adult stage at 20°C. Post-hoc one-way ANOVA on log10 transformed data with Bonferroni correction, with significance of pairs tested indicated at *P<0.05, ** P<0.01, ***P<0.001, ns =  not significant.

First hermaphrodites were down-shifted from 27°C to 20°C at the young adult stage, after all sperm were made, to test if temperature challenged sperm can fertilize a non-temperature challenged oocyte. Hermaphrodites down-shifted from 27°C to 20°C had a small but statistically significant increase in brood size when compared with hermaphrodites maintained at 27°C for all 5 isolates tested (Figure 6, Table 4). However, the brood size of down-shifted hermaphrodites was significantly lower than that seen in hermaphrodites raised and kept at 20°C. This is similar to the result seen with *C. elegans* isolates down-shifted from 25°C and *C. briggsae* isolates down-shifted from their thermal threshold of 30°C [3], [4]. When down-shifted hermaphrodites were mated with males to provide new sperm that should not be thermally compromised, there was a significant increase in the brood size; although again, the increase did not restore brood size back to the level seen in hermaphrodites continually kept at 20°C (Figure 6, Table 4). The small increase in brood size that is recovered upon down-shifting alone and the increased brood size recovered upon mating following the down-shift confirms previously seen effects on sperm function in hermaphrodites raised at high temperature that cannot recover because they cannot make new functional sperm [3], [4].

**Table 4:** Summary of brood sizes in hermaphrodite temperature-shift experiments.

Treatment	ED3072 mean brood size (SEM)	LKC34 mean brood size (SEM)	CB4856 mean brood size (SEM)	JU1171 mean brood size (SEM)	N2 mean brood size (SEM)
20°C	224.2 (6.9)	206.0 (9.5)	226.1 (6.9)	211.3 (6.6)	234.0 (7.9)
20°C+ males	297.5 (18.9)	332.5 (20.0)	253.3 (13.2)	215.5 (17.3)	329.0 (20.5)
27°C to 20°C	49.2 (6.1)	19.19 (2.5)	34.7 (6.2)	29.4 (3.6)	31.8 (4.6)
27°C to 20°C+ males	130.6 (14.6)	46.3 (5.9)	109.8 (11.2)	87.8 (7.4)	96.0 (1.3)
27°C	12.4 (1.8)	4.4 (0.7)	5.5 (1.1)	13.5 (1.4)	6.8 (1.3)
27°C+ males	37.6 (6.2)	8.2 (1.7)	9.2 (1.9)	35.4 (2.5)	23.4 (2.8)

Loss of function of sperm at high temperature does not preclude a loss of oocyte or oogenic germline function at high temperature that can recover upon down-shifting since oogenesis is ongoing. The small increase in brood size upon down-shifting without mating suggests that there may be an oogenic defect that does recover. To test this we mated hermaphrodites with males while maintaining them at 27°C. Experiments with up-shifting males to high temperature (Figure 5) demonstrated that in three of our isolates being tested there was no loss of fertility upon up-shifting. For the two isolates that did show some loss, more than half of the males were still fertile upon upshifting. Given that the individual hermaphrodites in these experiments were exposed to 6 males, any failure to see increased levels in brood size would indicate that there is a defect in the oogenic germ line. In two isolates, LKC34 and CB4856, there was no increase in brood size when hermaphrodites were mated at 27°C (Figure 6, Table 4). In the other three isolates there was an increase in brood size with mating at 27°C, but it was still significantly lower than the brood size seen in the same isolates when mated after down-shifting. Taken together these data indicate that there was an effect of temperature on both sperm and oogenic germline function, the relative effects of which differ between isolates. The complete lack of recovery of brood size upon mating at 27°C in LCK34 and CB4856 indicated that the high temperature effects on oogenic function in these isolates appears to be substantially stronger than in other isolates.

## Discussion

### *C. elegans* wild-type isolates show variation in brood size and fertility rates at 27°C that does not correlate with location of origin

Our studies have identified a set of three thermally tolerant and three thermally sensitive wild-type isolates that show significant differences in brood size at 27°C. Due to the fact that that there is strong correlation between the percentage of hermaphrodites that are fertile and the average brood size, it is likely that populations of thermal tolerant isolates would have a strong advantage at higher temperatures. Interestingly, two of the most thermal sensitive isolates were N2 and CB4856. These two isolates are some of the longest studied and most laboratory adapted of *C. elegans* isolates. Long-term culture at optimal temperature may lead to a loss of selective pressure for the ability to maintain fertility at more extreme temperatures. On the other hand, this may be a false correlation due to the small number of isolates analyzed. Other isolates that show very weak fertility at high temperature, including the thermal sensitive isolates LCK34, have been recently isolated and been carefully maintained to reduce lab adaptation. Either way, these sets of tolerant and sensitive isolates provide a means to investigate molecular and genetic pathways that underlie loss of fertility under temperature stress.

Although the *C. elegans* isolates analyzed here represent a diversity in their location of origin, there was no correlation found between brood size and location origin even when assayed at 27°C. This is in contrast to work done in Drosophilid species and *C. briggsae* [4], [9]. This is not entirely unexpected. *C. elegans,* unlike *C. briggsae,* does not show any clustering of either phenotypes or haplotypes by latitude or location of origin [12]. Work on other phenotypes including vulval development and thermotaxis behavior, also did not find any correlations between phenotypic outcome and location of origin/haplotype [12], [13]. Genome-wide SNP analysis of ∼200 wild-type isolates has shown that a recent selective sweep has reduced the genomic diversity in *C. elegans* isolates [14]. Interestingly one thermal tolerant isolate, ED3040, and one thermal sensitive isolate, LKC34, reside at closest proximity to each other on a neighbor-joining tree of 97 isotypes that come out of this work [14]. Thus, differences in brood size at high temperature could be due to newly acquired diversity at only a few genomic locations.

### Fertility in *C. elegans* demonstrates changes in plasticity across different temperatures

Waddington first proposed the idea that many traits, such as fertility, are canalized such that they demonstrate very little variation in response to genetic or environmental change [15]. These same traits can become more variable (plastic) even in genetically identical organisms under different conditions (for review see [16]). In the experiments presented here fertility shows some level of canalization at lower temperatures, the 20°C–24°C range, but becomes increasingly plastic at temperatures above this range. This can be seen in the smaller difference in brood size between 20°C–24°C when compared with the much greater change in brood size between 24°C–26°C, which represents a smaller change in temperature (Figure 1A). Additionally, variation in brood size within an individual isolate is lower at lower temperature ranges (20°C–24°C) but increases at more stressful temperatures (Figure 2). Many different studies have looked at how traits differ in plasticity across varying environments in both relatively outbred organisms, such as fish and snakes, and more genetically inbred systems, such as *Drosophila* and nematodes [4], [17]–[19]. For many of the traits examined there is a temperature range in which the trait shows low levels variation and temperature range(s), generally either at either extreme, where phenotypic variation increases.

The role of environmental canalization, low-level trait variation in response to environmental change, and its subsequent breakdown at environment extremes in the evolution of traits has been theorized about for decades [16], [20]. How then can increased plasticity of traits in response to fluctuating environments be selected for when there is a low level of genetic diversity to begin with, such as in *C. elegans* which has a low sequence diversity due to a recent selective sweep [14]? Recent work in *Drosophila* has provided a possible mechanism for how extremes in temperature can vary phenotypes in an epigenetic manor as a short-term response to environmental change that can be followed by a slower genetic change to fix the new phenotype [21]. In these studies it appears that Hsp90 may act as a capacitor for canalization of a trait following increased phenotypic plasticity (for review see [22]. In flies that have reduced Hsp90 function, either due to a weak allele or increase temperature, they exhibit much greater variation in phenotypes [21]. The individual phenotypes can be fixed, at least transiently, through a change in chromatin states, thus allowing for potential selection of that phenotype at the genetic level. Similar epigenetic mechanisms could be used in nematodes to respond to changes in global temperatures to allow for selection of genotypes that are adapted to have higher rates of fertility at more extreme temperatures.

### *C. elegans* males do not show robust recovery of fertility upon down-shifting to moderate temperatures

Male fertility, like that of hermaphrodites, was compromised when males were raised at 27°C. Notably, very little fertility was recovered when males were down-shifted to 20°C. This is in marked contrast to the ability of male fertility to recover in Drosophilids and mammals after a return to moderate temperature from increased temperature exposure. Approximately 80% of male *D. melanogaster* recover fertility after being raised at their thermal threshold for fertility and then down-shifted as young adults [23]. In the same study it was found that greater than 60% of males could recovery fertility after down-shifting even when raised at a temperature 1°C above their thermal threshold. In contrast, the highest percentage of males that showed recovery for the eight *C. elegans* isolates was 38% and half of the isolates assayed showed no recovery of male fertility. Since spermatogenesis occurs throughout the lifetime of male *C. elegans* and mating ability is maintained for at least 3 days past the young adult stage, the inability of males to recover viable sperm is surprising [24]. Further investigation into the functional loss of male fertility is needed to determine why males cannot recover fertility when moved to a moderate temperature.

There are many steps during spermatogenesis and mating that could be interrupted by high temperature exposure that lead to loss of fertility in males. The primary cause could be part of sperm formation or function including the formation of a functional germ line, the steps of spermatogenesis to form mature spermatids (non-mobile sperm), spermatid activation to form a pseudopod for movement, sperm movement into the spermatheca and sperm fertilization of the oocyte. Although these steps have not been fully investigated, preliminary analysis indicates that males do form fully developed germ lines with all of the appropriate stages of spermatogenesis (L. Petrella, unpublished data). Any later steps of sperm development and sperm function need to be investigated along with any changes in male behavior that could lead to an inability of males to mate with hermaphrodites. It has been shown that as male worms age their fertility decreases not due to loss of functional sperm but to loss of the ability to mate [24]. Thus, if males have a faster rate of aging at higher temperatures, their window of high mating efficiently may be reduced and this may play a role in the low level of male fertility recovery seen after down-shifting.

It seems unlikely that loss of male mating ability can be the sole reason that males lack fertility at high temperature. Given that hermaphrodite sperm function, which is not dependent on mating behavior, is also highly compromised at high temperature, it seems likely that there are also specific problems with sperm function that are occurring at high temperatures. Loss of male fertility in *Drosophila melanogaster* and mammals was found to be linked to a number of changes in sperm number and function. *D. melanogaster* males raised at the thermal threshold show decreased levels of motile sperm and these males appear to have problems with the steps of elongation and chromatin condensation in spermatogenesis [9]. In mammals, there is also a marked decrease in motile sperm, but in addition, *in vitro* fertilization studies have shown that motile sperm produced near the threshold for fertility have defects in the ability to penetrate the zona pellucida or activate oocytes [25]–[27]. *C. elegans* sperm differs from fly and mammalian sperm in that it moves by formation of a pseudopod and not by use of a flagellum but the ability of sperm to recognize and properly activate oocytes are important in all systems.

### 27°C represents a point of no-return in hermaphrodite germline function

As temperature increases, hermaphrodite brood size decreases gradually until 27°C, at which point there is a drastic drop in both the number of progeny and percentage of fertile worms in the population. At this thermal threshold for fertility, down-shifting hermaphrodites to a moderate temperature does not restore brood size, even after mating to provide functional sperm. This implies that some aspect of the oogenic germ line is unable to recover function. This is in contrast to experiments where worms were raised at the intermediate temperature of 25°C, where brood size is reduced but population fertility should still be near 100% [3]. At this temperature down-shifting with mating was able to fully restore brood size to those worms that were maintained at moderate temperatures their entire lives [3]. Preliminary analysis of hermaphrodite germ lines at 27°C showed that germline formation and gross anatomical structure look normal (L. Petrella, unpublished data). The primary cause of loss of fertility at high temperature appears to be dysfunction in some aspect of late germ cell function or that there is a signaling defect between the germ line and soma that is changed such that a fertilized embryo is not made. This hypothesis is based upon the fact that completely sterile worms have no evidence of unhatched embryos, while animals with very low numbers of progeny show very few unhatched embryos after 48 hours post lay (L. Petrella, unpublished data). While embryonic lethality could account for some level of the loss in progeny numbers it appears to be secondary to defects the germ line. The differences in recovery rates in different isolates after down-shifting could represent different levels of dysfunction in these germline pathways.

Loss of fertility as temperature increases has two aspects: from 20°–26°C there is a gradual decrease in brood size that is not accompanied by a decrease in the percentage of the population that is fertile, while from 26°C–27°C there is a dramatic decrease in both brood size and the percentage of fertile worms. Two scenarios could explain this phenomenon. The first is that there is some physiological pathway or process that gradually becomes dysfunctional as temperature increases but undergoes a catastrophic failure at a threshold. On the other hand, loss of each part of fertility (brood size and population fertility) may be due to dysfunction in two pathways, one that gradual fails and one that fails at the thermal threshold. One attractive model for this second scenario is that gradual loss of brood size is due to sperm dysfunction while the failure of the majority of worms to show any level of fertility is due to oocyte dysfunction. This is at least partially supported by the fact that worms raised at 25°C that are down-shifted and provided with fresh sperm fully recover brood size (Harvey 2007), while worms grown at 27°C that down-shifted and provided with fresh sperm do not. This could be explained by a difference in oogenic function between the two temperature treatments.

Work with temperature sensitive mutants in the N2 background has shown that there are a number of pathways that when mutated decrease the thermal threshold for fertility. It may be that some inherent temperature sensitivity in one of these pathways is the underlying cause of the loss in fertility in wild-type worms as temperatures increase. These pathways include the formation of P-granules, formation of the synaptonemal complex and germline specific small RNA pathways [28]–[30]. Interestingly, all of these pathways are specific to germ cells and do not play important roles in somatic function. Additionally, even if the individual genes are not conserved, similar pathways are conserved in both mammalian and Drosophilid germ lines, making them attractive targets for dysfunction at high temperature. Having multiple thermal tolerant and thermal sensitive isolates will allow us to investigate whether there are genetic changes in these pathways that could underlie differences in brood sizes at high temperature.

## Methods and Materials

### Worm maintenance

Worms were cultured using standard techniques [31]. 22 wild-type isolates from a variety of latitudes and origins of locations were used (Table 1). The following isolates (origin of location) were received from the *Caenorhabditis* Genomics center. MY1 (Lingen, Germany), MY2 (Roxel, Germany), MY3 (Roxel, Germany), CB4932 (Tauton, UK), TR389 (Madison, WI), JU1171 (Concepcion, Chile), JU1172 (Concepcion, Chile), JU1652 (Montevideo, Uruguay), AB3 (Adelaide, Australia), CB4507 (Palm Canyon, CA), ED3046 (Ceres, South Africa), JU258 (Ribeiro Frio, Madeira), ED3040 (Johannesburg, South Africa), CB4856 (Hawaii, USA), DL238 (Manuka, HI USA), LKC34 (Madagascar), DL200 (Addis, Ababa, Ethiopia), ED3077 (Nairobi, Kenya), ED3072 (Limuru, Kenya). The N2 Bristol strain used was that kept and maintained in the Strome lab at UC Santa Cruz. HT1593 or SS1057 *unc-119(ed3)* strains used in male mating experiments were from the CGC or Strome lab, respectively. All strains were maintained at 20°C on OP50 *E. coli* unless shifted for experimentation.

### Hermaphrodite brood size at continuous temperature

In order for the entire development of the germ line to take place at the assayed temperature and to ensure that hermaphrodites being tested did not experience starvation, a condition that can alter life-history traits, P0 hermaphrodites were raised at 20°C until the late-L4 stage. 8–12 L4 animals were then shifted to the assay temperature overnight. The following day P0 animals were transferred to new plates for 4–6 hours to obtain a cohort of F1 animals that had grown continuously at the assay temperature. After 2–3 days, depending on temperature, late-L4 F1 animals were cloned out as a single worm to a plate and transferred daily to a fresh plate until reproduction ended. After the F1 was removed, F2 embryos were allowed to develop to the L2–L4 stage and counted. All plates with progeny for a single individual were summed to reach total brood size. Any worms that burrowed, crawled away and desiccated or died before the average age of loss of egg laying were removed from analysis. Each isolate was tested at each temperature with at least two replicates of 10–12 worms (giving at least 20 worms per temperature per isolate). Experiments were always done on the same shelf, in the same location in the incubator to reduce internal incubator differences and a temperature probe was located near the sealed box to verify incubator temperature. Within a single experiment, plates of different isolates were intermingled to diminish the effect of any thermal gradients within the incubator.

Statistical analysis of brood size was done on fertile animals, brood sizes of zero were excluded. In order to account for non-normality and heterogeneity of variances, data were Box-Cox transformed prior to the building of the model and post-hoc analysis. For analysis a multiple linear regression model was constructed in R (http://www.r-project.org) [32] with brood size described as a function of rearing temperature (20°C, 24°C, 26°C, 27°C), genotype and temperature × genotype interaction. Including latitude of origin, region of origin (Equatorial, Tropical and Temperate), haplotype (based on Andersen et al., 2011), average summer high temperature at location of origin, or average winter high temperature at location did not strengthen the model. Analysis using an ANCOVA test in R gave exactly the same results. Post-hoc analysis using ANOVA was done to compare isolates at each temperature using Box-Cox transformed brood size, with Bonferroni correction on resulting *P*-values in Prism 6 (GraphPad). To compare population fertility rates and lifetime brood size, linear regression analysis was performed in Prism 6 (GraphPad).

### Male experiments

Male lines of individual isolates were established by heat-shock of L4 hermaphrodites and then maintained through mating. For all experiments, a single male at the late L4/young adult stage was placed on a plate with 5–6 *unc-119(ed3)* hermaphrodites. After ∼48 hours, males were then moved to a new plate containing fresh *unc-119(ed3)* hermaphrodites. Plates were scored for the presence of non-unc progeny ∼72 hours post-male presence. For graphical representation of male temperature-shift experiments see Figure S1. Males lines and *unc-119* strains were maintained at 20°C. For up-shift experiments late L4/young adult males were moved from 20°C to 27°C with adult *unc-119* hermaphrodites. Males were moved to a new plate with newly up-shifted *unc-119* hermaphrodites as described above. The plates were maintained at 27°C until scored. For 27°C and 27°C down-shift experiment, P0 L4/young adult males and L4 hermaphrodites from a specific isolate were up-shifted together from 20°C. After two days at 27°C, all P0 males were removed so that they would not be mistaken for F1 males. On day three or four, single F1 late L4/early adult males were cloned out with *unc-119* hermaphrodites grown at 20°C and either maintained at 27°C or down-shifted to 20°C. Similar to experiments described above, males were moved to a new plate with new *unc-119* hermaphrodites and scored for non-unc progeny. Differences in the percentage of males able to produce cross progeny were analyzed using a Fisher's exact test in Prism 6 (GraphPad).

### Mated hermaphrodite and temperature-shift experiments

For graphical representation of hermaphrodite temperature-shift experiments see Figure S2. For 20°C experiments: Isolate lines were maintained at 20°C. Individual young adult hermaphrodites where moved to a plate either alone or in the presence of 5 L4/young adult males of the same isolate. After 48 hours worms were moved to a fresh plate at 20°C, if mated 3 fresh young adult males were added at this time. After 18–24 hours progeny on the first plate was scored as larvae. After 48 hours of the P0 on the second plate, larvae on the second plate were scored. The number of progeny was then combined as the 96 hour brood size.

For 27°C and down-shifted experiments: 8–12 L4 hermaphrodites were up-shifted from 20°C to 27°C. After ∼12 hours the P0s were moved as a cohort to a second plate. Both plates were maintained at 27°C for 2–3 days until they were enriched for F1 young adult animals. At this point individual young adult hermaphrodites were move to a plate either alone or containing 5 male worms of the same isolate that were raised to the L4/young adult stage at 20°C. Plates were either maintained at 27°C until scoring for progeny or down-shifted and maintained at 20°C. All proceeding steps were the same as those described for the 20°C experiments. Resulting brood sizes were log10 transformed and then analyzed by ANOVA, with Bonferroni correction on resulting *P*-values using Prism 6 (GraphPad).

## Supporting Information

Figure S1 **Scheme for male temperature-shift experiments.** Blue line represents F1 male whose fertility is being tested, green line represents *unc-119* hermaphrodites mated to F1 males. P0 parents are not shown on graph but were grown post L4 stage at the same temperature as the shown F1 progeny. Time points: A) Late L4/young adult males are cloned to individual plates containing 5–6 *unc-119* hermaphrodites. B) After ∼48 hours males are moved to a fresh plate with new *unc-119* hermaphrodites. C) Original plates from time point A are scored for the presence of non-unc progeny. D) Plates set up from time point B are scored for the presence of non-unc progeny. # Hermaphrodites raised at 20°C were added to cloned out male plates at this time.
(TIFF)

Figure S2 **Scheme for hermaphrodite temperature-shift experiments.** Green line represents F1 hermaphrodite whose fertility is being tested, blue line represents males of the same isolate mated to F1 hermaphrodites. P0 parents are not shown on graph but were grown post L4 stage at the same temperature as the shown F1 progeny. Time points: A) Young adult hermaphrodites are cloned to individual plates either alone or with 5 young adult males. B) After ∼48 hours both hermaphrodites alone or hermaphrodites and males are moved to a fresh plate and 3 fresh young adult males were added. C) All progeny on original plates from time point A are counted. D) All progeny on original plates from time point A are counted. # males raised at 20°C were added to cloned out hermaphrodite plates at this time.
(TIFF)
