# Supplementary figures and images for: Natural Variants of C. elegans Demonstrate Defects in Both Sperm Function and Oogenesis at Elevated Temperatures
Source: PLoS One. 2014 Nov 7;9(11):e112377. doi: 10.1371/journal.pone.0112377 (PMC4224435; doi:10.1371/journal.pone.0112377)

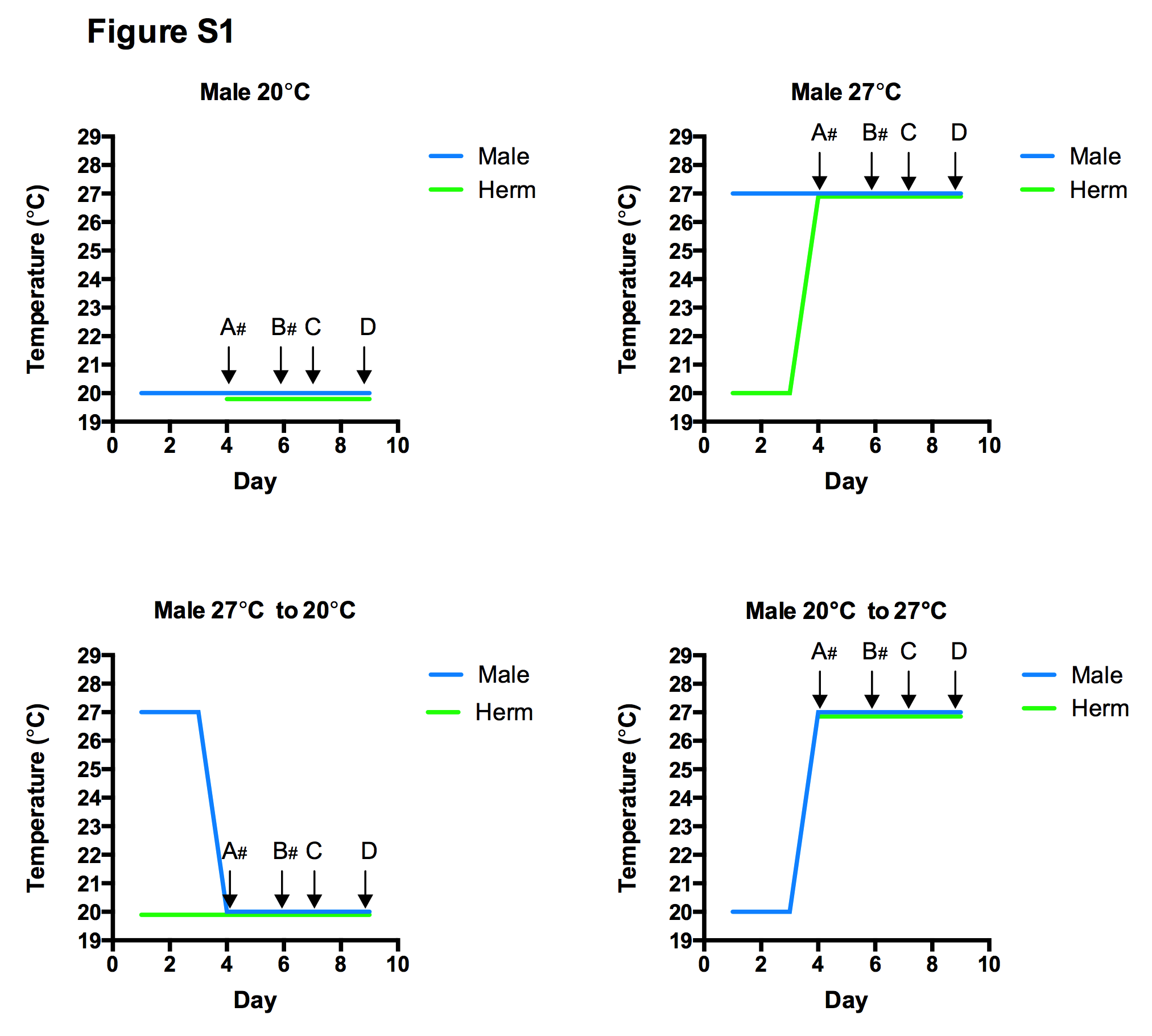

Supplement: Figure S1 — Scheme for male temperature-shift experiments. Blue line represents F1 male whose fertility is being tested, green line represents unc-119 hermaphrodites mated to F1 males. P0 parents are not shown on graph but were grown post L4 stage at the same temperature as the shown F1 progeny. Time points: A) Late L4/young adult males are cloned to individual plates containing 5–6 unc-119 hermaphrodites. B) After ∼48 hours males are moved to a fresh plate with new unc-119 hermaphrodites. C) Original plates from time point A are scored for the presence of non-unc progeny. D) Plates set up from time point B are scored for the presence of non-unc progeny. # Hermaphrodites raised at 20°C were added to cloned out male plates at this time. (TIFF) [file pone.0112377.s001.tiff]

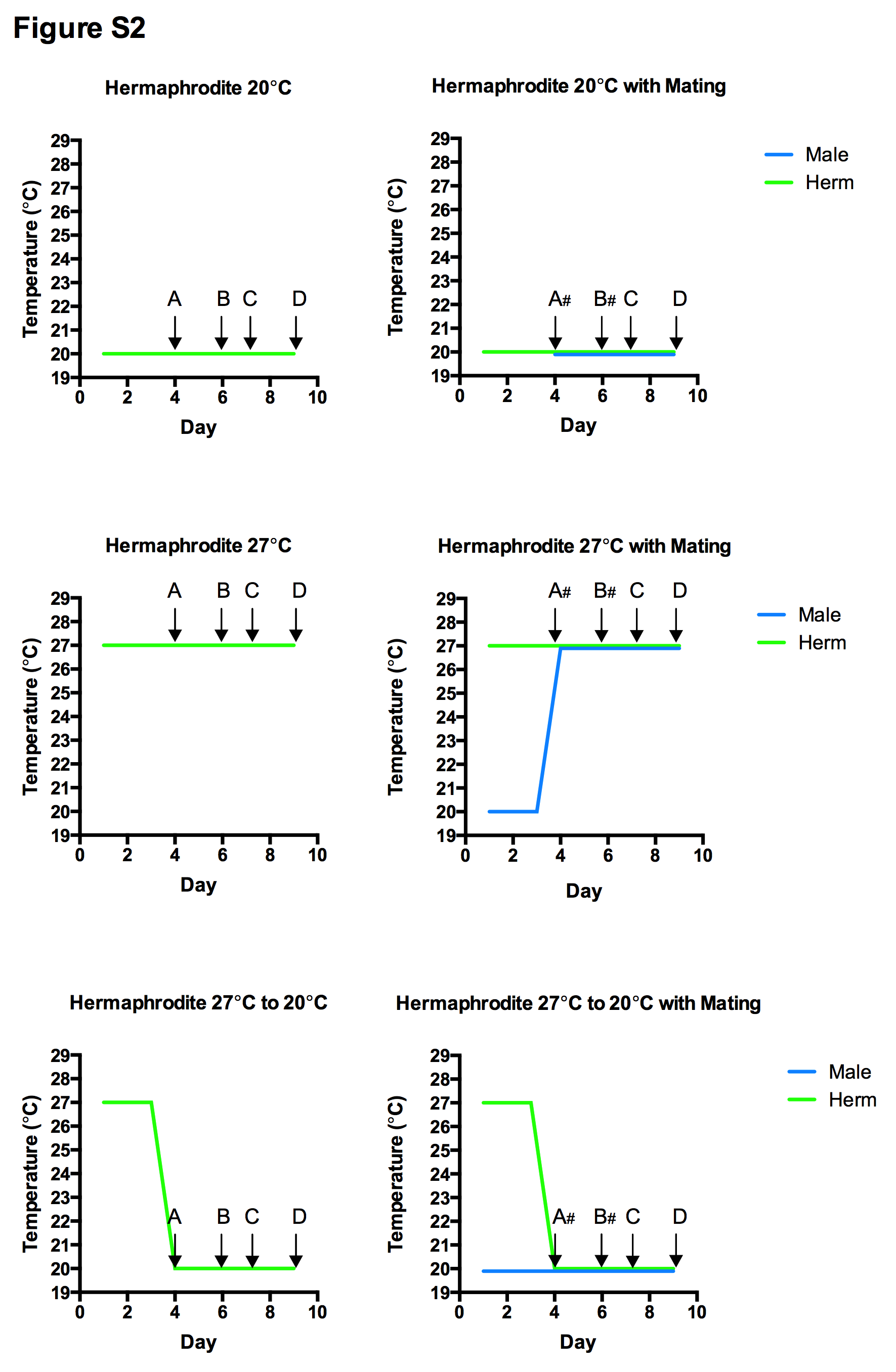

Supplement: Figure S2 — Scheme for hermaphrodite temperature-shift experiments. Green line represents F1 hermaphrodite whose fertility is being tested, blue line represents males of the same isolate mated to F1 hermaphrodites. P0 parents are not shown on graph but were grown post L4 stage at the same temperature as the shown F1 progeny. Time points: A) Young adult hermaphrodites are cloned to individual plates either alone or with 5 young adult males. B) After ∼48 hours both hermaphrodites alone or hermaphrodites and males are moved to a fresh plate and 3 fresh young adult males were added. C) All progeny on original plates from time point A are counted. D) All progeny on original plates from time point A are counted. # males raised at 20°C were added to cloned out hermaphrodite plates at this time. (TIFF) [file pone.0112377.s002.tiff]
